# Clinical course, costs and predictive factors for response to treatment in carpal tunnel syndrome: the PALMS study protocol

**DOI:** 10.1186/1471-2474-15-35

**Published:** 2014-02-07

**Authors:** Christina Jerosch-Herold, Lee Shepstone, Edward CF Wilson, Tony Dyer, Julian Blake

**Affiliations:** 1 Faculty of Medicine and Health Sciences, University of East Anglia, Norwich, UK; 2 Norwich Clinical Trials Unit, University of East Anglia, Norwich, UK; 3 Cambridge Centre for Health Services Research, University of Cambridge, Cambridge, UK; 4 Department of Neurophysiology, Norfolk and Norwich University Hospitals NHS Foundation Trust, Norwich, UK

## Abstract

**Background:**

Carpal tunnel syndrome (CTS) is the most common neuropathy of the upper limb and a significant contributor to hand functional impairment and disability. Effective treatment options include conservative and surgical interventions, however it is not possible at present to predict the outcome of treatment. The primary aim of this study is to identify which baseline clinical factors predict a good outcome from conservative treatment (by injection) or surgery in patients diagnosed with carpal tunnel syndrome. Secondary aims are to describe the clinical course and progression of CTS, and to describe and predict the UK cost of CTS to the individual, National Health Service (NHS) and society over a two year period.

**Methods/Design:**

In this prospective observational cohort study patients presenting with clinical signs and symptoms typical of CTS and in whom the diagnosis is confirmed by nerve conduction studies are invited to participate. Data on putative predictive factors are collected at baseline and follow-up through patient questionnaires and include standardised measures of symptom severity, hand function, psychological and physical health, comorbidity and quality of life. Resource use and cost over the 2 year period such as prescribed medications, NHS and private healthcare contacts are also collected through patient self-report at 6, 12, 18 and 24 months. The primary outcome used to classify treatment success or failures will be a 5-point global assessment of change. Secondary outcomes include changes in clinical symptoms, functioning, psychological health, quality of life and resource use. A multivariable model of factors which predict outcome and cost will be developed.

**Discussion:**

This prospective cohort study will provide important data on the clinical course and UK costs of CTS over a two-year period and begin to identify predictive factors for treatment success from conservative and surgical interventions.

## Background

Carpal Tunnel Syndrome (CTS) is the commonest peripheral entrapment neuropathy which causes pain, tingling, numbness and weakness in the distribution of the median nerve of the hand. It is bilateral in 55-65% of cases [1] and is a significant contributor to impaired hand function and work disability. In the UK in 2000 the annual age standardised rate per 100,000 was 87.8 for men and 192.8 for women [2]. There are no estimates of the cost of CTS to UK society or the NHS, however societal costs to the USA of patients undergoing decompression surgery were estimated at over $2bn in 1995 [3]. More recently in 2007, the income loss per CTS patient over a period of 6 years was estimated at $45,000-89,000 compared with controls (upper extremity fracture and dermatitis) [4].

Treatment options which are supported by high quality evidence are corticosteroid injection for mild to moderate symptoms [5,6] and surgical decompression in moderate to severe cases [7]. Surgery rates in 1995 in the UK were 71 per 100,000 [8] and rising to 90/100,000 a decade later [9]. Although steroid injection is an effective and relatively safe first line of treatment, its benefit beyond 8 weeks has not been demonstrated [5]. A UK study by Latinovic found that 31% of patients proceeded to surgery within 1 year of initial presentation to their General Practitioner [2]. A study in the Netherlands found that only 25% of patients had a good outcome at 1 year after steroid injection [10]. Similarly, Meys et al. [11] reported that 67% of patients treated by injection required surgery by 1 year. Surgical rates vary from country to country and depend on referral criteria. The introduction of 'low priority procedure’ thresholds by NHS trusts in England may account for lower surgery rates in the UK compared to the US or Europe.

The treatment algorithm currently used by most NHS Trusts in England is to refer patients to surgery only when symptoms are severe and disabling or when steroid injections have failed. Where injection fails to resolve symptoms, this prolongs the duration of symptoms and associated disability as well as incurring direct and indirect costs. As yet it is not possible to predict which patients will obtain long-term relief from steroid injection and which will require surgical decompression. Longer duration of symptoms and greater severity of symptoms are also associated with poorer surgical outcomes although the evidence seems equivocal [12].

Surgical decompression is effective in most patients, however reported success rates vary. An analysis of pooled results from 209 studies, representing 32,936 operations, found that 75% of patients considered themselves improved, much better or cured [13]. Misdiagnosis and surgical errors such as incomplete decompression are the most common reasons for failure. The answer to the question of what factors predict a good surgical outcome has so far eluded us.

The authors of a recent systematic review of surgical and non-surgical treatments concluded that future research needed to focus on 'prognostic studies which lead to better patient characterisation and the identification of predictive factors that indicate likely response to specific treatments’ [14]. Although a number of studies have explored prognostic factors, these have been conducted either retrospectively or in the context of randomised trials which has limitations [15]. To date no prospective cohort study of prognostic factors has been undertaken. The value of high quality prognostic research is that it leads to a better understanding of disease progression, better targeting of effective treatments that mitigate progression and allows more reliable information about the risk of a negative outcome to be communicated to patients [15]. A cohort study of prognostic factors would be the first necessary step towards the development and further external validation of clinical prediction rules. This work would also address the need to focus on outcomes that matter to patients as outlined in the NHS Outcomes Framework [16] by collecting patient-reported outcomes on one of the commonest elective surgical procedures in the upper extremity.

### Aims and objectives

The primary aim of this cohort study is to identify prognostic indicators of outcome for surgical and conservative treatment by corticosteroid injection in patients presenting for nerve conduction tests with symptoms of carpal tunnel syndrome, managed according to current best practice and followed up for up to 2 years.

Specific objectives are:

1) To identify baseline clinical, psychological and sociodemographic predictors for response to treatment by injection and surgical decompression in patients diagnosed with CTS.

2) To describe the course and progression of CTS in treated and untreated patients over 2 years from diagnosis.

3) To describe and identify factors that predict the cost of CTS to the NHS and society over a period of 2 years.

4) To pilot a web-based system for routine collection of clinical baseline and patient-reported outcomes in patients undergoing treatment for CTS.

## Methods/Design

### Design

A prospective observational cohort study of patients diagnosed with CTS through clinical examination and history and confirmed by nerve conduction studies. Data on putative prognostic factors and outcomes of corticosteroid injection and surgery for CTS will be collected over a 2 year period.

### Study setting

In Norfolk approximately 1200 patients per annum are referred with suspected carpal tunnel syndrome to clinical neurophysiology at the Norfolk and Norwich University Hospitals NHS Foundation Trust (NNUH). Nerve conduction studies (NCS) are done on both hands to confirm a clinical diagnosis, objectively grade the severity of compression and make treatment recommendations based upon it. Referrals come from primary care e.g. general practitioner, community physiotherapist, and secondary care e.g. orthopaedics, rheumatology and plastic surgery.

### Inclusion criteria

Adults aged 18 years and over who attend for NCS in the Department of Neurophysiology and are confirmed to have CTS of at least mild severity (grade ≥ 1 on Padua scale [17]) based on a combination of NCS results and clinical diagnosis, in at least one hand and for which either no treatment is recommended at present or conservative or surgical treatment is recommended. Patients with idiopathic as well as secondary CTS due to diabetes or hypothyroidism will be included.

### Exclusion criteria

•Patients with unilateral CTS who have had carpal tunnel surgery in that hand in the last 12 months.

•Pregnancy and up to and including 12 months postpartum due to the often transient nature of CTS.

•Serious co-morbidity e.g. malignancy, severe mental illness and where the GP considers the patient to be vulnerable and for whom participation would be otherwise detrimental.

•Other symptomatic upper limb mononeuropathies, vibration induced conditions, radiculopathy or thoracic outlet syndrome.

•Any other sensory or motor disturbances affecting the upper limb such as stroke, multiple sclerosis, nerve injury.

•Unable to read or write English.

### Recruitment procedure

Potentially eligible patients will be identified by the consultant neurophysiologist based on the clinical history and NCS results. The patients will be informed verbally about the current study and given an information pack containing an invitation to participate, a screening questionnaire and consent form. Patients will be asked to read through these in their own time at home and a telephone number for the study coordinator is provided if patients have any further questions. Patients who wish to participate will be asked to complete and return the consent form and screening questionnaire in an enclosed stamped addressed envelope within 2 weeks. Those who do not wish to participate will be asked to still complete the screening questionnaire which does not require disclosure of any personally identifiable information. Their data will be used to compare the consenters with non-consenters and assess external validity of the study. Those patients returning a valid signed consent form will be contacted by the study coordinator via the telephone who will verify that they meet eligibility criteria and also to verify that patients have given fully informed consent. In order to maximise response rates a reminder letter will be sent by the consultant neurophysiologists to all patients who were deemed eligible and given a study invitation pack within 1 week of attendance at the department of Neurophysiology. Each patient will only be enrolled into the study once even where CTS presents bilaterally.

### Care pathway

The NCS report from the Neurophysiologist will determine the severity of CTS and include treatment recommendations. The clinical management of patients will be at the discretion of the consultant clinical neurophysiologist or referring clinician informed by local guidelines for commissioning and best practice guidelines. Patients who are eligible for the study will fall into three categories: i) no further treatment recommended or required (watch and wait), ii) recommended for conservative treatment by splinting and/or corticosteroid injection; iii) recommended for surgical decompression.

i) No further treatment – patients in this group are expected to have very mild symptoms and NCS confirming a mild compression. They either do not require any conservative treatment or have declined the offer of conservative treatment such as corticosteroid injection.

ii) Conservative treatment – patients in this group are expected to have mild to moderate CTS based on NCS results and will be offered steroid injection and splints.

iii) Surgical treatment – patients in this group are expected to have moderate to severe CTS based on NCS results and self-reported symptom severity. These patients are likely to meet the current thresholds for surgical referral which are: referral to secondary care if symptoms are not resolved to patient’s satisfaction after 6 months of conservative treatment from date of 1st consultation with physician or if neurological deficit is present, i.e. sensory blunting or weakness of thenar abduction (APB).

During the 2 year follow-up, patients may undergo different treatments depending on the course of the CTS. For example a patient may be recommended not to have treatment when first enrolled however 6 months later symptom severity and associated disability may have increased necessitating conservative treatment. Or a patient may be treated with corticosteroid injection initially and progress to surgery in the course of the 2 years. Patients may also seek or be offered other treatments including alternative therapies.

All types of treatments received for CTS of either hand will be recorded by patients through 6 monthly self-report. To assist patients in the recall of treatments received a 6 months diary card will be issued in which they can record date, type, dosage and out of pocket expenditures for any treatments for CTS.

### Questionnaire data

Data on putative predictive factors will be collected with a study specific questionnaire through a combination of standardised measures and purposely designed questionnaires:

Symptom severity will be assessed with the 6-item short form of the Boston Carpal Tunnel Questionnaire, the CTS-6 [18].

Hand functional status will be assessed with three subscales of the Michigan Hand Questionnaire (MHQ) which capture unilateral and bilateral hand function, activities of daily living and work performance [19].

Psychological status has been identified as a predictor of outcome in several studies and will be measured using the standardised Hospital Anxiety and Depression scale (HADs) [20].

Health-related quality of life and health utility will be assessed by the EQ-5D-3 L (http://www.EuroQol.org). Results of the EQ-5D-3 L will be converted into utilities by applying UK specific preference weights to the health profiles. QALYs accrued will be calculated by integrating utility with respect to time. QALYs accrued in year two will be discounted by 3.5%.

Comorbidity will be assessed using a self-administered standardised Comorbidity Questionnaire developed by Sangha et al. [21].

Additionally, at baseline other clinical and sociodemographic factors will be collected through self-report including age, sex, ethnicity, work status and type of work, smoking status, alcohol use, household income.

Results from NCS will be requested from the neurophysiologist once patients have consented and been enrolled into the study. Median nerve sensory nerve action potential (amplitude and velocity) for digit III and distal motor latency (amplitude and velocity) will be recorded. Padua’s criteria [17,22] for classifying CTS severity according to the neurophysiology results will be used to grade severity from 1 to 6 (mild to extremely severe).

### Resource use and cost

Patients will be asked to complete a set of resource use questions asking about prescribed medications, primary, secondary and tertiary NHS and private healthcare contacts, alternative therapies and over the counter medicines purchased and time off work due to CTS since the last follow-up. The questionnaire completed at baseline will pertain to resource use in the three months prior to commencement of the study. Following completion of the baseline questionnaire and each follow-up (except final follow-up), patients will be issued with a 'resource use diary’ in the form of a card with tick-boxes and space for notes for patients to complete over the interim time period [23]. This will be used as an aide memoire when completing the resource use sections of the questionnaires.

To verify the patient-self-reported data, at the 2 year follow-up the General Practitioner of a random sample of at least 10% of patients will be contacted to confirm numbers of primary care contacts for CTS, whether the patient is still receiving treatment for CTS, details of drug and dosage where corticosteroid injection has been given, and other drugs prescribed for CTS. Records of surgery performed will also be extracted and compared with patient reported data.

### Outcomes

The primary outcome used to classify treatment successes and failures will be patient-reported global assessment of outcome (GAO) at 2 years using a 5 point ordinal scale: worse = 1, unchanged = 2, slightly better = 3, much better = 4, completely cured = 5. A grade of 3 or above will be defined as treatment success. Secondary outcomes include patient-reported clinical symptoms (CTS-6), functioning (MHQ), psychological health HADs, quality of life (EQ-5D) and resource use.

### Data collection methods

All patients enrolled into the study will be asked to complete a questionnaire at baseline and again at 6, 12, 18 and 24 months. Given the increasing proportion of patients who have access to the internet it is possible to collect these data online using secure encrypted web-based systems which are a cost-effective alternative to paper questionnaires. At initial enrolment patients will be asked if they have access to the internet and given the option to complete all follow-up questionnaires by mail or electronically. Data entry will be via a web-based system provided by Norwich Clinical Trials Unit Data Management team. Any individual, whether study staff or participant entering data online will gain access to the website using a unique username and password. Participants will only have access to their own data. Once a participant has confirmed the submission of data, they will not be able to change it. The database is built using MS SQL Server and the website and associated software is built using MS ASP.NET. The database and website reside on the Norwich CTU secure server at University of East Anglia. Data from patients who opt to return questionnaires via the post will be entered by a research associate. On receipt of a completed online or paper questionnaire participants will be sent a thank you card and a diary to help them record information about treatments received and out of pocket expenses incurred as a result of the carpal tunnel syndrome for the forthcoming 6 months.

### Sample size

An estimated 1200 patients are seen at the Norfolk and Norwich University Hospital (NNUH) each year with suspected carpal tunnel syndrome. Of these, at least 85% (around 1020) will be confirmed as having CTS through NCS indicating a mild or worse compression. Assuming a recruitment rate of 75%, which is not unreasonable, given previous experiences, we would expect to recruit around 765 CTS subjects per annum, i.e. 1530 over the two year recruitment period. It is estimated that around 30% (460) will go to surgery immediately and 70% (1070) will be treated with corticosteroid injection. Of the latter, it is expected that, by two years follow-up, two thirds will have been referred for surgery, whereas the remaining third (about 355) will be considered a success. There is no standard agreed method for calculating sample sizes or statistical power for logistic regression models. However, following the approach by Demidenko [24] a sample size of 1070 would confer 90% statistical power, assuming a 33% success rate, to detect an increase in success with an odds ratio of 1.51 for an explanatory binary variable with an even split in classification (i.e. an equal number of subjects in each group). For an uneven split, this odds ratio would be smaller. Should recruitment be less than expected, a sample size of 535 (i.e. half that expected) would confer 90% power to detect an odds ratio of 1.79.

### Analysis

The primary outcome (global assessment of outcome) will be modelled using a binary logistic regression model. Predictors will be considered on a univariate basis and those found to have a statistically significant effect on the outcome (at the 10% level) will be entered simultaneously into a multivariable model. A backwards deletion approach based upon change in likelihood will be used to remove those predictors with no significant independent effect. An ordinal logistic regression model will also be considered based upon the proportional odds assumption using the original 5 point ordinal scale as the outcome. However, as the assumption is often difficult to verify, this will be considered as a secondary analysis.

Secondary outcomes will be analysed in a similar fashion using regression models with link functions and error terms appropriate to the distribution of the outcome of interest.

#### Cost of illness analysis

The resource use data will be used to perform a longitudinal estimate of the cost of illness of carpal tunnel syndrome in the subject cohort over a period of two years from the perspectives of the NHS and society (defined as the sum of NHS and patient out of pocket costs and morbidity-related lost productivity). The total cost of each patient will be calculated as the sum of resource quantities multiplied by relevant unit costs extracted from standard NHS databases (e.g. NHS reference costs and Unit Costs of Health and Social Care). Unit cost sources will be from a common price year, to be determined at time of analysis. Costs incurred in year two will be discounted at 3.5% as per HM Treasury guidelines.

Regression models will be developed as described above to predict cost over two years according to a number of prognostic variables, e.g. symptom severity or functional status at baseline, age, and socioeconomic status. The analysis will make appropriate adjustments to take account of missing and censored data where necessary. Where there are systematic differences between patient self-reported data and primary care records, a secondary analysis will be conducted adjusting primary care cost for any observed bias.

### Project timetable

Recruitment to the study began in July 2013 and is due to be completed by July 2015. The two-year follow-up is anticipated to be completed in July 2017.

### Ethical approval

The PALMS study was approved by the East of England/Norfolk Research Ethics Committee (REC reference 13/EE/0106) on 22 April 2013.

## Discussion

CTS is the most common nerve entrapment disorder of the upper limb which can lead to considerable hand disability and sickness absence in those working, yet little is known about the cost of CTS to the individual, NHS or society. A range of effective treatment options are available including conservative and surgical interventions. However it is not known which patients are more likely to benefit from what treatment. This prognostic study will provide important information about what baseline factors may predict a good long-term response to conservative treatment and which patients may ultimately require surgery. It is an important first step in building a multivariable model, which can be externally validated in future studies. The value of high quality prognostic research is that it leads to a better understanding of disease progression, better targeting of effective treatments that mitigate progression and allows more reliable information about the risk of a negative outcome to be communicated to patients [15].

We were faced with a number of design issues that arose during development of the study, not least in the choice of outcome measures. A review of the literature was undertaken to identify those factors which have been shown to be predictive of outcomes for surgical and conservative treatments. Although a number of studies have explored predictive factors these have been conducted either retrospectively, are based on small sample sizes or have been done in the context of randomised trials which has limitations. Katz et al. [25] studied 241 patients enrolled in a community based study of outcomes for CTS treatment. 78% completed follow-up at 18 months. In a bivariate analysis higher pre-operative symptom severity and functional limitations (measured on Boston Carpal Tunnel Questionnaire [26]), lower mental health status and lower physical health status (measured by SF-12) were associated with worse outcome after surgery. Work-related variables associated with a worse outcome after surgery were being in receipt of workers’ compensation and where an attorney was involved, heavy manual labour and those using repetitive forceful exertions. Linear regression identified greater pre-operative functional limitation, tobacco and alcohol use, worse mental health status, prominent day pain and bilateral symptoms as predictors of worse outcome. Those more intensively exposed to keyboard activities had better symptom status. A study by Meys [11] at al examined prognostic factors for response to corticosteroid injection in a prospective cohort study of 118 patients in the Netherlands. Successful outcome was defined as no need for further treatment within 1 year of injection. 67.4% proceeded to surgery by 1 year. Predictors of successful outcome from steroid injection were a lower Boston symptom severity score, lower median nerve ultrasonic cross-sectional area and lower median nerve swelling ratio.

Other putative factors which have been identified in the literature include duration of symptoms, severity of paraesthesia at night [27], constant paraesthesia and older age [28]. Whilst these studies do not offer any definitive answers they have been useful in identifying putative predictors which should be included in our study.

We therefore chose to measure CTS severity, hand functional status, psychological status, overall health related quality of life, co-morbidities, as well as health service resource use and out of pocket costs.

Symptom severity in CTS is normally assessed using the symptom severity subscale of the standardised, disease-specific and patient-reported Levine scale [26] (also called the CTS questionnaire or Boston Carpal Tunnel Questionnaire). It is validated for patients with CTS and has been widely used in trials for conservative and surgical treatment for CTS [29]. Recently a shortened version has been developed by Atroshi et al. [18] and its psychometric properties assessed using both factor analysis and item-response theory (RASCH analysis). It contains 6 items rather than 11 items. We therefore elected to use this version.

Hand functional status can be assessed using the 8-item subscale of the Levine scale, however it has been shown that the items are not specific enough to CTS [18] and other measures of hand and arm disability have been developed since, including the Disabilities of the Arm, Shoulder and Hand (DASH) [30] and Michigan Hand Questionnaire (MHQ) [19]. A limitation of the DASH questionnaire is that the score can be greatly affected by comorbid lower limb disability [31] and it does not generate a separate score for each hand. The MHQ is the only scale which measures function for the left and right hands separately as well as bilateral activities. The MHQ is a 57- item hand-specific outcome questionnaire comprising 6 subscales that measure i) unilateral and bilateral hand function, ii) activities of daily living, iii) pain, iv) work performance, v) aesthetics and vi) patient satisfaction. The MHQ has been shown to be responsive to change in patients undergoing surgical treatment for CTS (effect sizes ranging from 0.6 to 0.9) [32]. The MHQ pain subscale substantially overlaps with the pain questions in the CTS-6 scale and the MHQ aesthetics subscale is not relevant in CTS. The MHQ satisfaction subscale overlaps with the primary outcome measure, the 5 point global assessment of change. We therefore elected to use the remaining 3 subscales of the MHQ in this study, that is, hand function, activities of daily living and work performance.

Resource use data will be collected by self-reported questionnaire. Such data are at risk of recall bias, [33] although patients in general practice appear to be reasonably accurate in estimating GP contacts over a six month period [34]. Use of a diary as an aide memoire will help ensure fidelity of the data. However, to further verify the patient-self-reported data, we will contact at least 10% of patients’ GPs to confirm primary care activity as described above. Records of surgery performed will also be extracted and compared with patient reported data, although evidence suggests that primary care records are a less than ideal source of validation of secondary care activity [35]. Patient recall for secondary care activity is considered fairly reliable [33].

We chose to include patients with diabetes within the study. Patients with diabetes may or may not also have a diabetic neuropathy. However as this is very difficult to differentially diagnose from the CTS, it would be difficult to apply an exclusion criterion for those with diabetic neuropathy reliably. There will also be a large proportion of patients with diabetes and CTS but no diabetic neuropathy. Thus excluding those with diabetes may significantly limit the number of patients who can be recruited, as well as potentially affecting the generalizability of the results. Moreover by including these patients in the analysis it will be possible to examine the effect of diabetes as a comorbid factor on outcome.

We chose to recruit patients through clinical neurophysiology. This has two advantages: firstly, the diagnosis of CTS will be made through a combination of good clinical examination, history taking and NCS, therefore reducing the risk of treatment failures due to misdiagnosis. Secondly, it provides a single point of recruitment for patients referred from primary and secondary care from a large catchment area in Norfolk/Suffolk. Nevertheless, although it was initially planned as a single centre study, initial recruitment data indicates that the response rate is lower than anticipated and further departments of neurophysiology within the UK will be included subject to local research governance approval.

This prospective cohort study will provide information on the course and cost of CTS to the individual, the health service and society as well as identify predictive factors for a good outcome from conservative and surgical treatment. It may guide future development of clinical care pathways for CTS and provide important information to service users and providers on the likely expected outcome from treatment.

## Competing interests

The authors declare that they have no competing interests.

## Authors’ contributions

CJH conceived the original idea and applied for funding. All authors were involved in the design of the study protocol and drafting of the manuscript. All authors have read and approved the final manuscript.

## Pre-publication history

The pre-publication history for this paper can be accessed here:

http://www.biomedcentral.com/1471-2474/15/35/prepub
